# Supplementary material for: Genomic Legacies of Ancient Adaptation Illuminate GC-Content Evolution in Bacteria
Source: Microbiol Spectr. 2022 Dec 13;11(1):e02145-22. doi: 10.1128/spectrum.02145-22 (PMC9927291; doi:10.1128/spectrum.02145-22)
Supplement: Supplemental file 1 — Fig. S1 to S18 and Table S2. Download spectrum.02145-22-s0001.pdf, PDF file, 2.1 MB [file spectrum.02145-22-s0001.pdf]

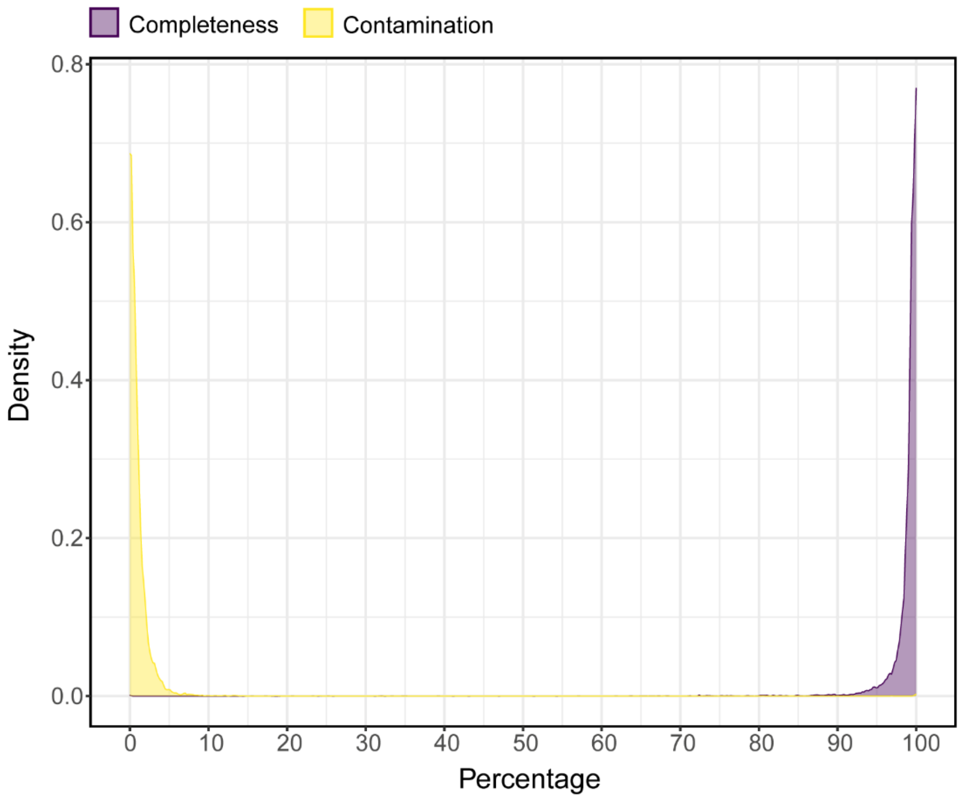

FIG S1 Quality distribution of all bacterial representative genomes. Purple represents genome completeness and yellow represents genome contamination.

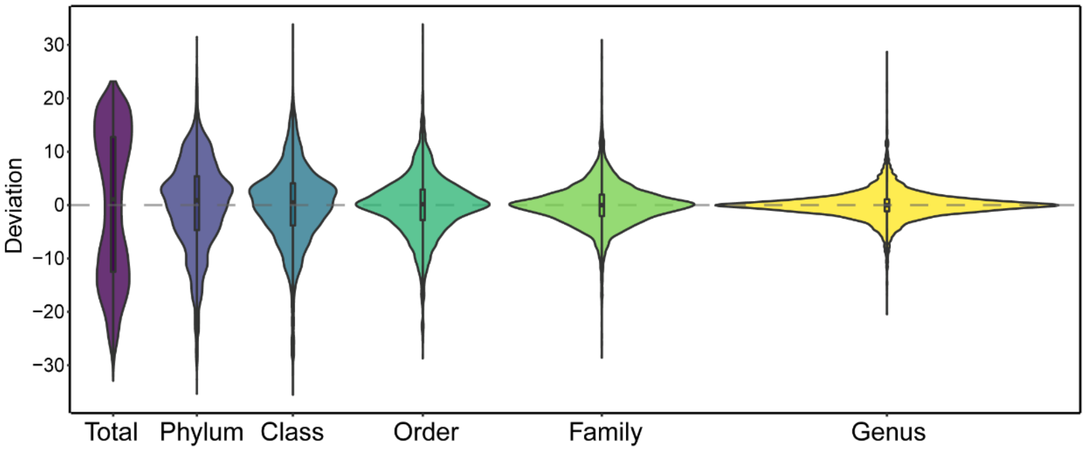

FIG S2 Within-taxon variation of genomic GC-content at different taxonomic levels. The deviation represents the difference between GC content value and the average value within a specific taxon.

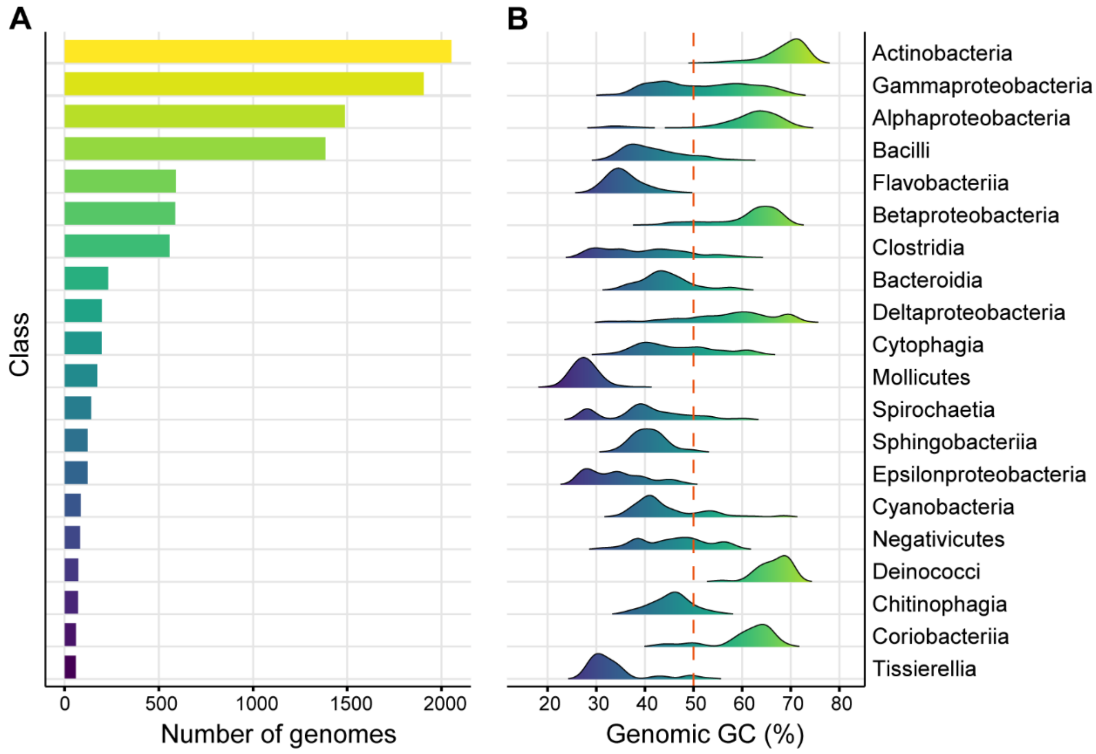

FIG S3 Distribution of the genomic GC at the class level. (A) Distribution of the genomic GC content of classes with more than 50 representative genomes. (B) Number of representative genomes of classes in (A).

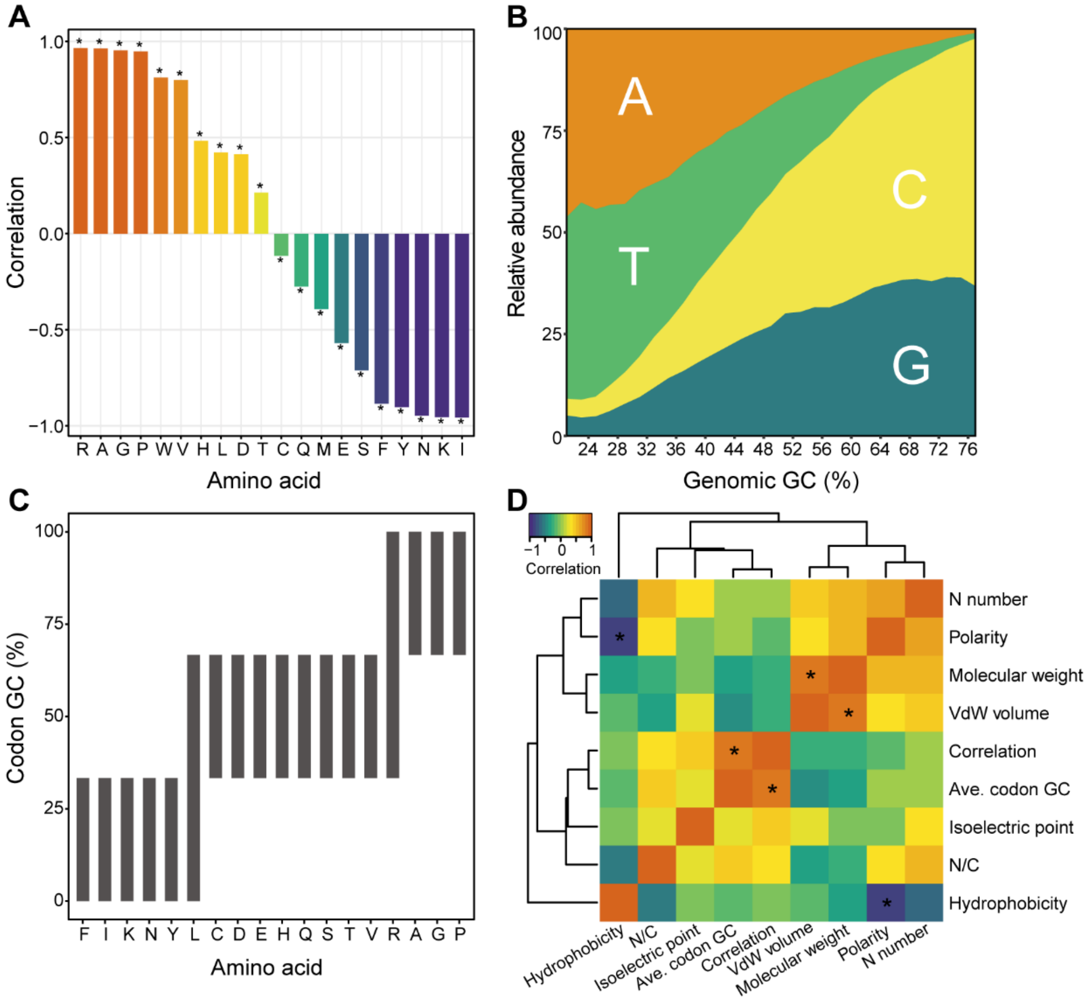

FIG S4 Relationships between the genomic GC, codon usage and amino acid usage. (A) Correlations between the genomic GC and the abundance of amino acids. (B) Abundance of codons with different base in the third position is correlated to the genomic GC content. Only quartets (i.e., the codons of A, G, P, V and T) are analyzed. (C) GC-content range of the codons of each amino acid. (D) Pairwise correlation tests between the correlation with the genomic GC, the average codon GC and chemical properties of each amino acid. Asterisks in (A) and (D) indicate adjusted p-value < 0.01.

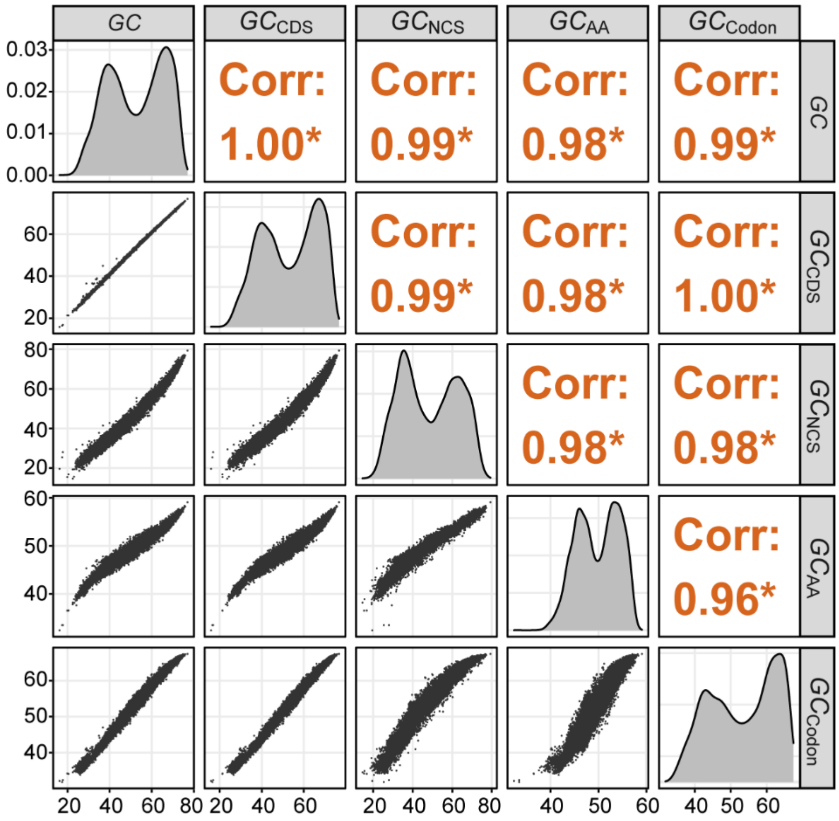

FIG S5 High-consistency in the GC content variation. Pairwise correlation scores between the GC content genome wide (*GC*), GC content of coding sequences (GC_{CDS}), GC content of non-coding sequences (GC_{NCS}), and GC content contributed by amino-acid usage (GC_{AA}) and synonymous codon usage (GC_{Codon}) are shown.

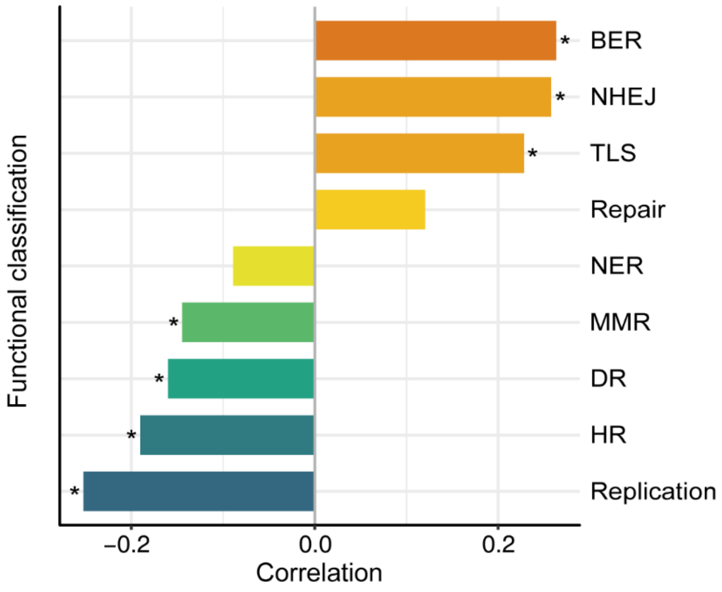

FIG S6 Correlations between the genomic GC and DRR-related pathways using the phylogenetic independent contrast (PIC). Asterisks indicate adjusted p-value < 0.05.

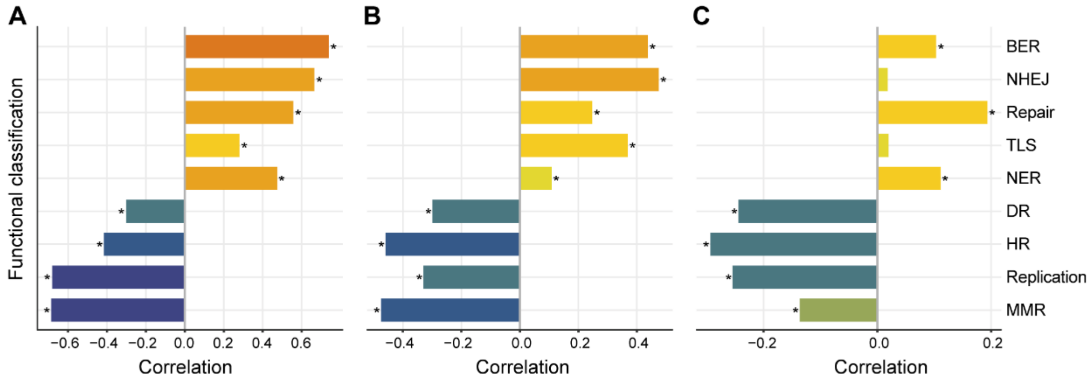

FIG S7 Correlations between the genomic GC-content and DRR-related pathways. (A) Correlations between the genomic GC and DRR-related pathways in the Terrabacteria clade (B) Correlations between the genomic GC and DRR-related pathways in the Proteobacteria clade. (C) Correlations between the genomic GC and DRR-related pathways in the FCB & PVC clade.

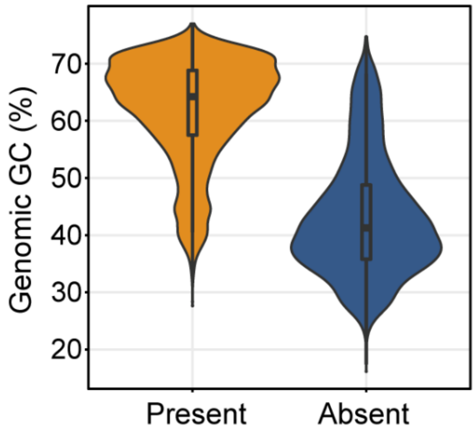

FIG S8 Comparison of the GC content of genomes with and without YbbN.

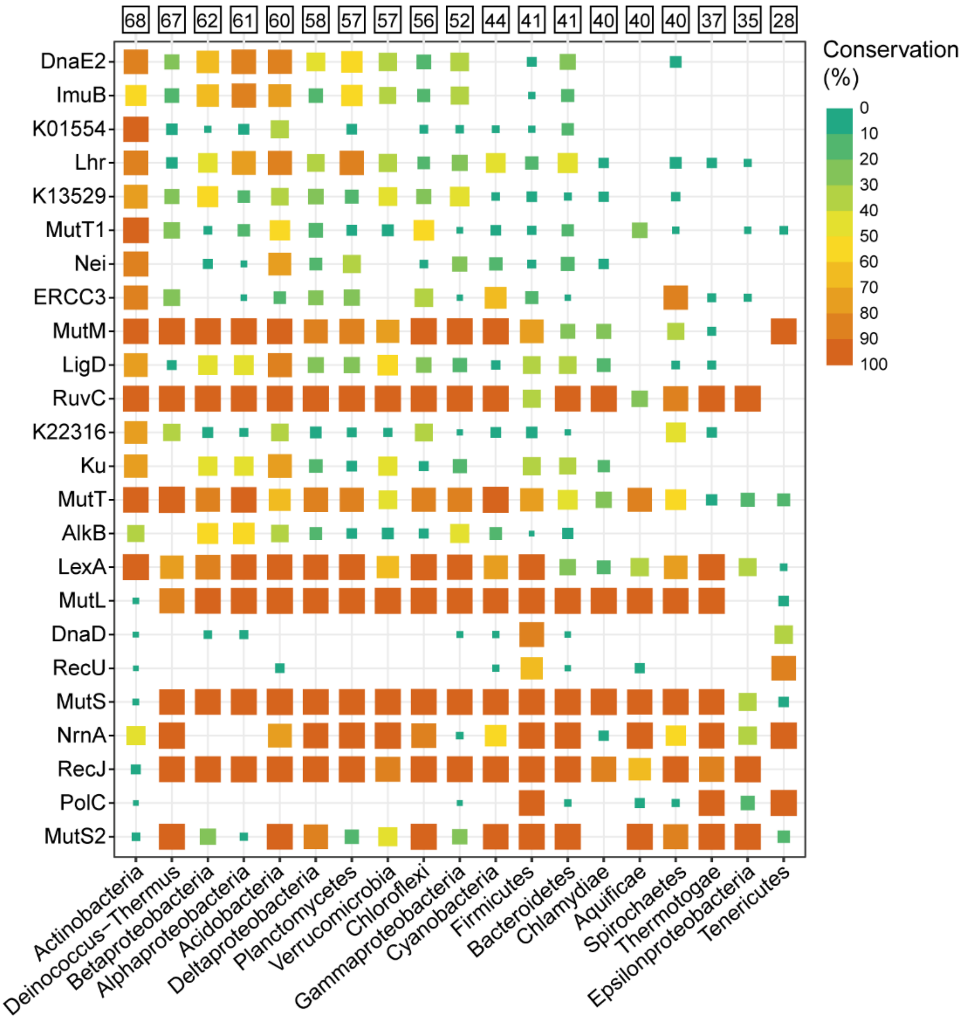

FIG S9 Conservation of DRR-related KOs shown in Fig. 3C in major clades of bacteria. The number in square above the plot shows the average genomic GC content of each clade.

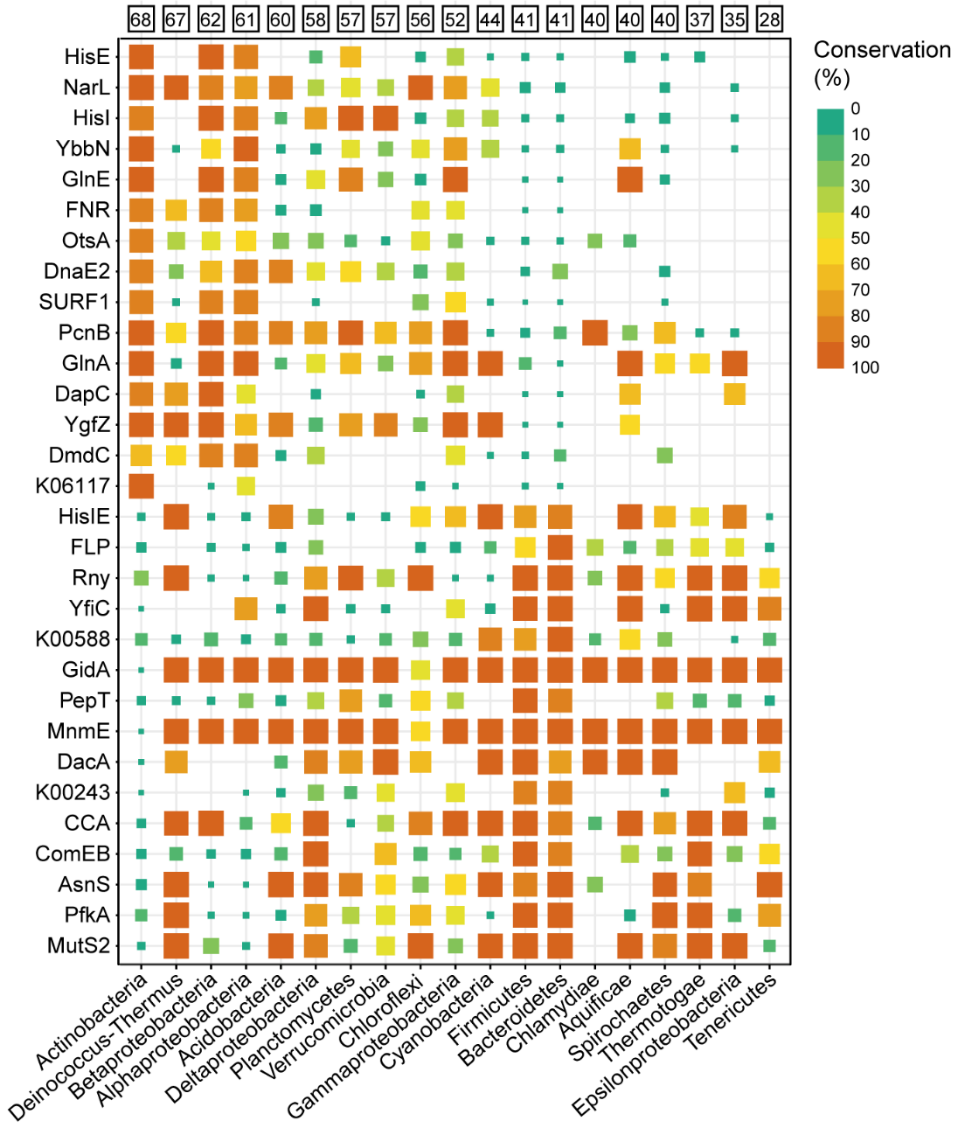

FIG S10 Conservation of the highly correlated KOs shown in Fig 4A in major clades of bacteria. The number in square above the plot shows the average genomic GC content of each clade.

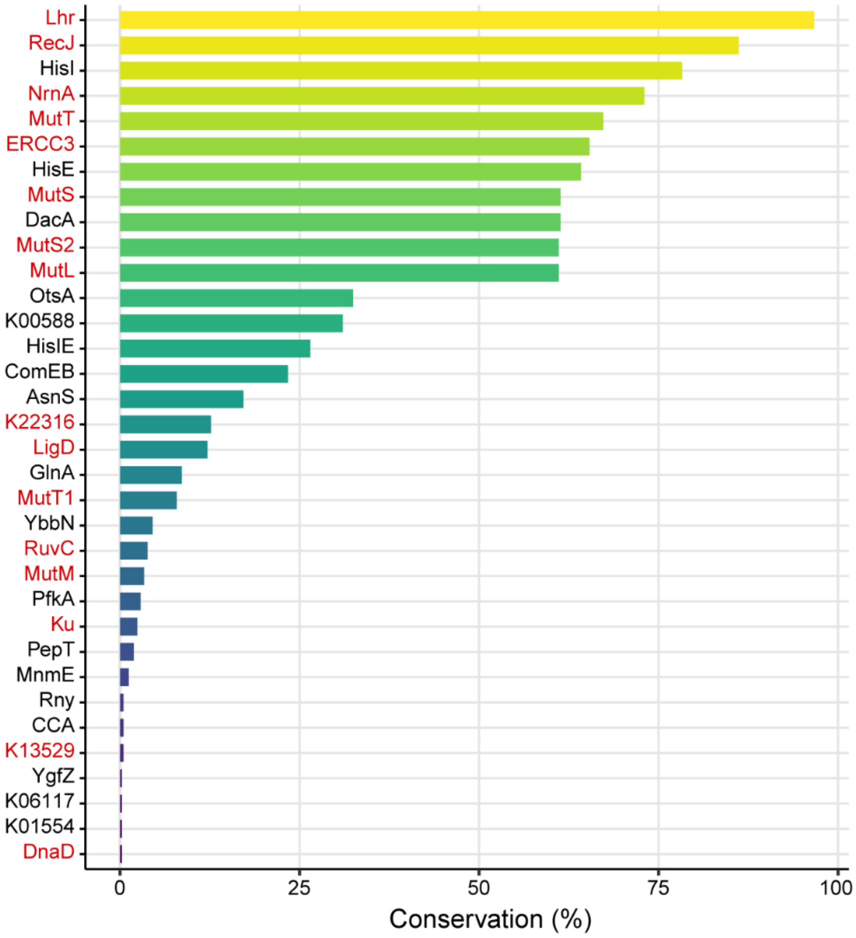

FIG S11 Conservation of KOs shown in Fig. 3C and Fig. 4A in archaea. The names of DRR-related KOs are shown in red. Completely missing KOs are neglected.

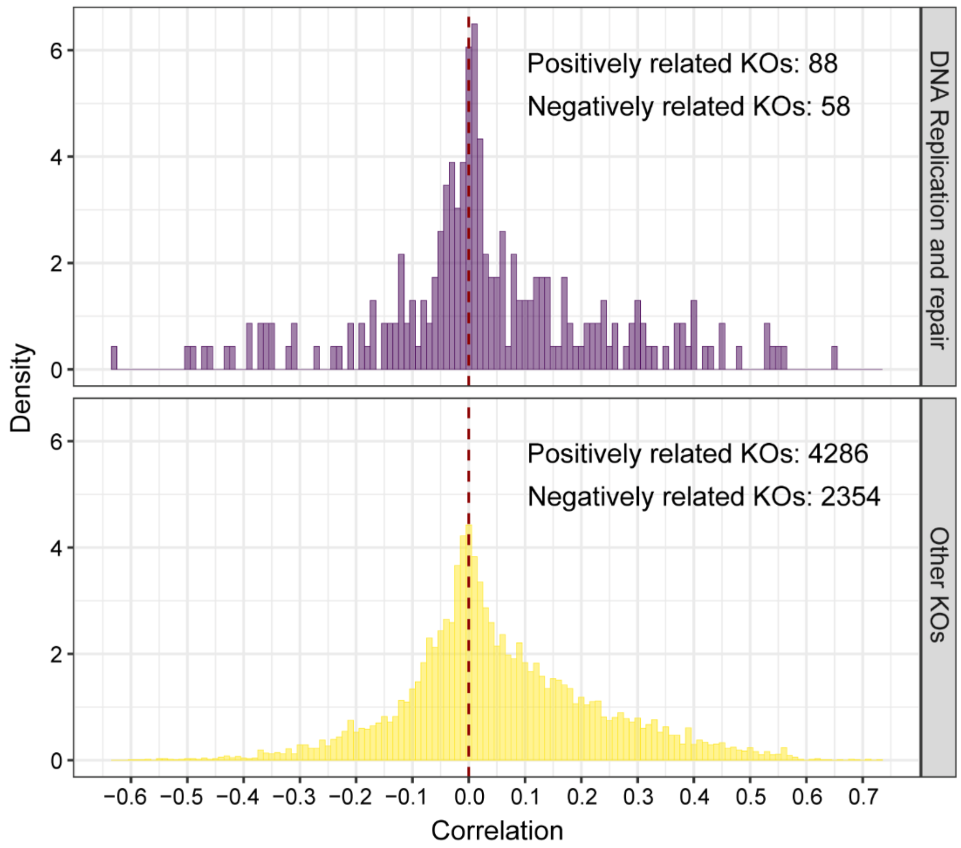

FIG S12 Distribution of the correlations between the genomic GC and annotated KOs. DRR-related KOs and the others are displayed separately.

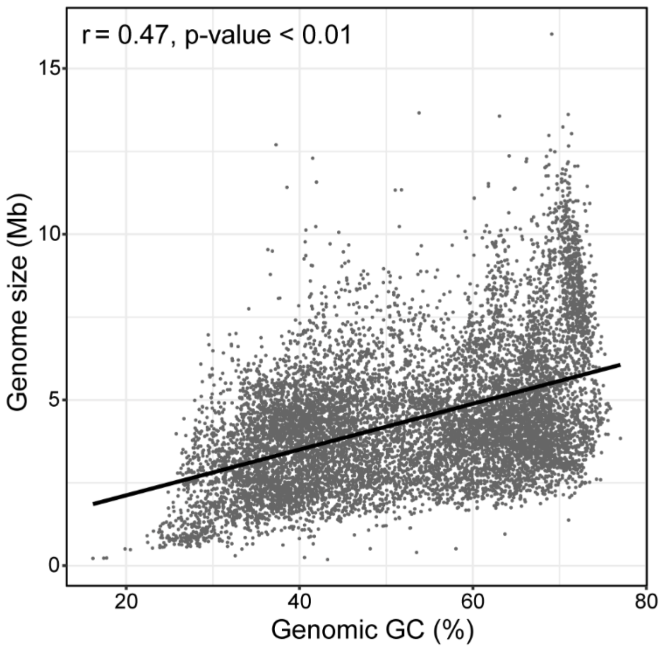

FIG S13 Linear regression analysis between the genomic GC content and genome size. Pearson's correlation coefficient and p-value are labeled on the plot.

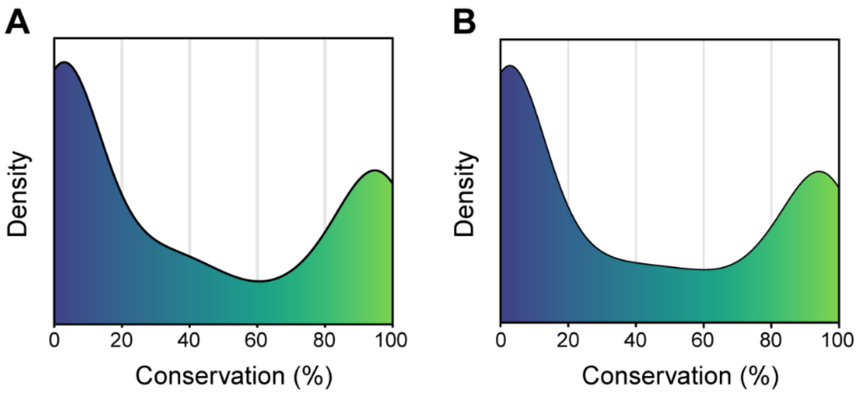

FIG S14 Distribution of the conservation of highly correlated KOs. (A) Distribution of the conservation data shown in Fig. S9. (B) Distribution of the conservation data shown in Fig. S10.

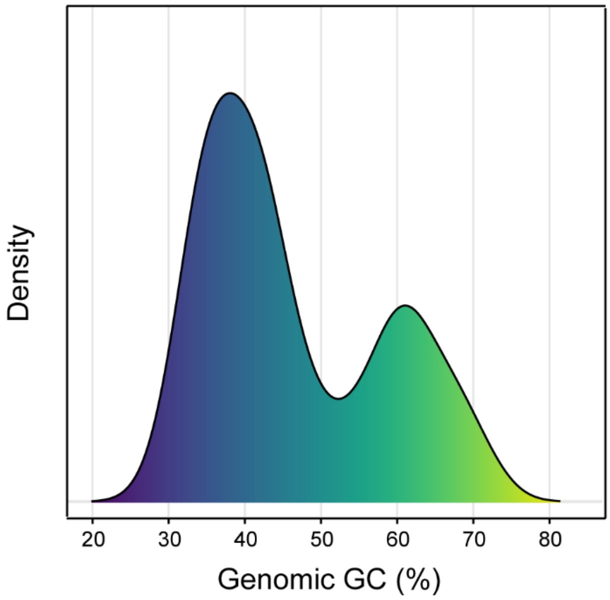

FIG S15 Distribution of the genomic GC of psychrotolerant species.

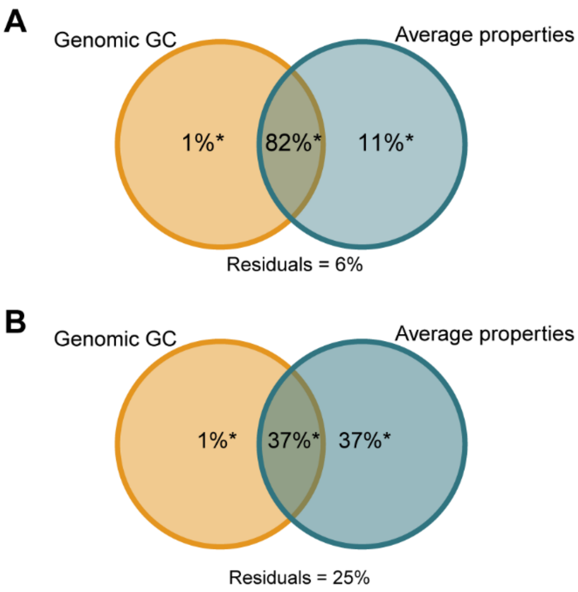

FIG S16 Variance partitioning analysis of the amino acid composition. (A) Variance partitioning analysis of the amino acid composition of all bacterial proteins. (B) Variance partitioning analysis of the amino acid composition of the conserved regions in 16 ribosomal proteins. Asterisks beside the percentages indicate adjusted p-value < 0.01.

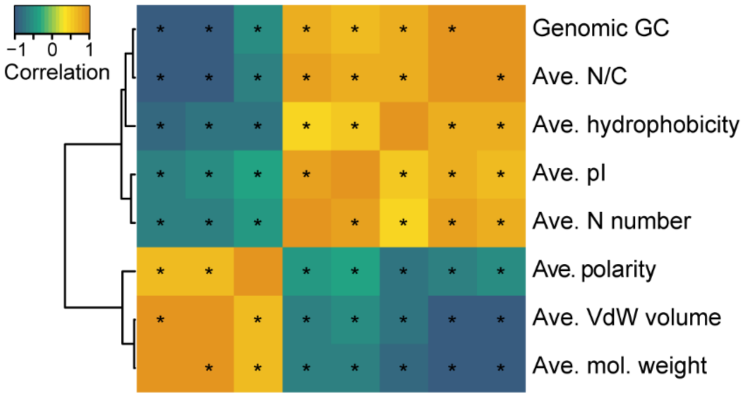

FIG S17 Correlations between the genomic GC and average amino acid properties in bacteria. Asterisks in the plot indicate adjusted p-value < 0.01.

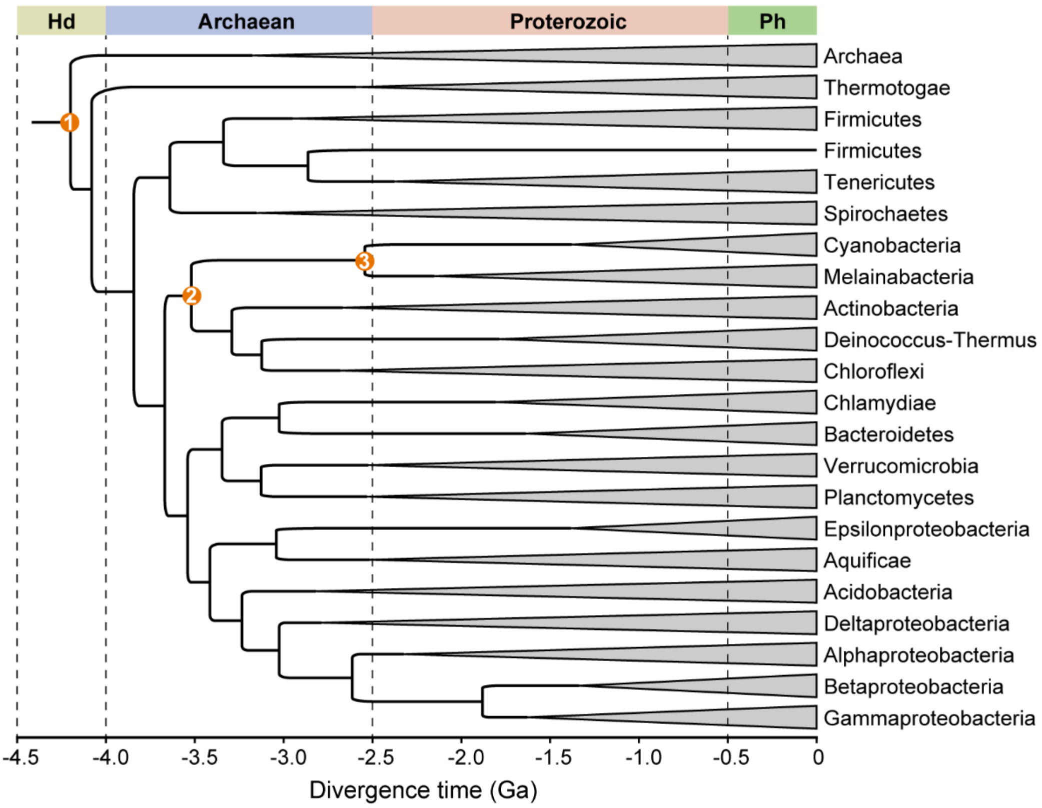

FIG S18 Molecular dating of the phylogenetic tree of bacteria. Orange circles on the tree indicate the temporal constrains (node 1: < 4.52 Ga; node 2: > 3.225 Ga; node 3: 2.5-2.6 Ga).

TABLE S2 Function of KOs correlated to the genomic GC content

KO	Protein	Function in previous studies
K01523	HisE	Involve in the biosynthesis of histidine (1)
K07684	NarL	Function as a nitrate/nitrite response regulator (2)
K01496	HisI	Involve in the biosynthesis of histidine (1)
K05838	YbbN	Function as a chaperone of β-clamp to cope with heat stress (3)
K00982	GlnE	Regulate the glutamine synthetase in response to nitrogen limitation (4)
K00528	FNR	Participate in the defense against oxidative damage (5)
K00697	OtsA	Involve in the synthesis of trehalose in response to heat, cold and osmotic stresses (6)
K14998	SURF1	Regulate the assembly of cytochrome c oxidase involved in aerobic respiration (7)
K00970	PcnB	Accelerate the mRNA degradation according to growth conditions (8)
K20712	GlnA	Participate in the biodegradation and metabolism of xenobiotics (9)

K14267	DapC	Involve in the biosynthesis of lysine (10)
K06980	YgfZ	A tRNA-modifying protein which can increase the resistance to oxidative stress (11)
K20035	DmdC	Involve in the assimilation of dimethylsulphoniopropionate produced by marine phytoplankton (12)
K06117		Involve in the recycle and catabolism of glycerophospholipid (9)
K11755	HisIE	Involve in the biosynthesis of histidine (1)
K21562	FLP	Function as an anaerobic regulatory protein in response to oxidative stress (13)
K18682	Rny	Interact with glycolytic proteins by processing the mRNA of *gapA* operon (14)
K15460	YfiC	Modify valine-specific tRNA to promote growth in combating hyperosmotic and oxidative stress (15)
K00588		Involve in the biosynthesis of secondary metabolites (16)
K03495	MnmG	Involve in the modification of tRNAs (17)
K01258	PepT	Catalyze the release of free amino acids for nutritional utilization of tripeptides (18)

K03650	MnmE	Involve in the modification of tRNAs (17)
K18672	DacA	Convert ATP or ADP into the c-di-AMP which regulate various cellular processes (19)
K00243		Uncharacterized protein
K00974	CCA	Add the nucleotides CCA onto the 3' end of tRNA precursors (20)
K01493	ComEB	Involve in the uptake of exogenous DNA under nutrient starvation (21)
K01893	AsnRS	Catalyze the specific aminoacylation of tRNA Asn with asparagine (22)
K00850	PfkA	Be required for the utilization of more carbon sources (23)

References

1. Del Duca S, Chioccioli S, Vassallo A, Castronovo LM, Fani R. 2020. The role of gene elongation in the evolution of histidine biosynthetic genes. Microorganisms 8:732.
2. Yoshida M, Ishihama A, Yamamoto K. 2015. Cross talk in promoter recognition between six NarL-family response regulators of Escherichia coli two-component system. Genes Cells 20:601-612.

3. Le H-T, Gautier V, Kthiri F, Kohiyama M, Katayama T, Richarme G. 2011. DNA replication defects in a mutant deficient in the thioredoxin homolog YbbN. Biochem Biophys Res Commun 405:52-57.

4. Carroll P, Pashley CA, Parish T. 2008. Functional analysis of GlnE, an essential adenylyl transferase in Mycobacterium tuberculosis. J Bacteriol 190:4894-4902.

5. Krapp AR, Tognetti VB, Carrillo N, Acevedo A. 1997. The role of ferredoxin-NADP+ reductase in the concerted cell defense against oxidative damage. Eur J Biochem 249:556-563.

6. Jiang Y, Chen X-M, Liu Y-J, Li Y-T, Zhang H-H, Dyson P, Sheng H-M, An L-Z. 2010. The catalytic efficiency of trehalose-6-phosphate synthase is effected by the N-loop at low temperatures. Arch Microbiol 192:937-943.

7. Mick DU, Wagner K, van der Laan M, Frazier AE, Perschil I, Pawlas M, Meyer HE, Warscheid B, Rehling P. 2007. Shy1 couples Cox1 translational regulation to cytochrome c oxidase assembly. EMBO J 26:4347-4358.

8. Zhang X-X, Liu Y-H, Rainey PB. 2010. CbrAB-dependent regulation of pcnB, a poly(A) polymerase gene involved in polyadenylation of RNA in Pseudomonas fluorescens. Environ Microbiol 12:1674-1683.

9. Schenzle A, Lenke H, Spain JC, Knackmuss HJ. 1999. 3-Hydroxylaminophenol mutase from Ralstonia eutropha JMP134 catalyzes a Bamberger rearrangement. J Bacteriol 181:1444-1450.

10. Velasco AM, Leguina JI, Lazcano A. 2002. Molecular evolution of the lysine

biosynthetic pathways. J Mol Evol 55:445-449.

11. Waller JC, Ellens KW, Hasnain G, Alvarez S, Rocca JR, Hanson AD. 2012. Evidence that the folate-dependent proteins YgfZ and MnmEG have opposing effects on growth and on activity of the iron-sulfur enzyme MiaB. J Bacteriol 194:362-367.

12. Reisch CR, Stoudemayer MJ, Varaljay VA, Amster IJ, Moran MA, Whitman WB. 2011. Novel pathway for assimilation of dimethylsulphoniopropionate widespread in marine bacteria. Nature 473:208-211.

13. Gostick DO, Griffin HG, Shearman CA, Scott C, Green J, Gasson MJ, Guest JR. 1999. Two operons that encode FNR-like proteins in Lactococcus lactis. Mol Microbiol 31:1523-1535.

14. Commichau FM, Rothe FM, Herzberg C, Wagner E, Hellwig D, Lehnik-Habrink M, Hammer E, Völker U, Stülke J. 2009. Novel activities of glycolytic enzymes in Bacillus subtilis. Mol Cell Proteomics 8:1350-1360.

15. Golovina AY, Sergiev PV, Golovin AV, Serebryakova MV, Demina I, Govorun VM, Dontsova OA. 2009. The yfiC gene of E. coli encodes an adenine-N6 methyltransferase that specifically modifies A37 of tRNA. RNA 15:1134-1141.

16. Do C-T, Pollet B, Thévenin J, Sibout R, Denoue D, Barrière Y, Lapierre C, Jouanin L. 2007. Both caffeoyl Coenzyme A 3-O-methyltransferase 1 and caffeic acid O-methyltransferase 1 are involved in redundant functions for lignin, flavonoids and sinapoyl malate biosynthesis in Arabidopsis. Planta

226:1117-1129.

17. Shi R, Villarroya M, Ruiz-Partida R, Li Y, Proteau A, Prado S, Moukadiri I, Benítez-Páez A, Lomas R, Wagner J, Matte A, Velázquez-Campoy A, Armengod ME, Cygler M. 2009. Structure-function analysis of Escherichia coli MnmG (GidA), a highly conserved tRNA-modifying enzyme. J Bacteriol 191:7614-7619.

18. Cha MH, Yong WM, Lee SM, Lee YS, Chung IY. 2000. The biochemical and molecular characterization of recombinant Bacillus subtilis tripeptidase (PepT) as a zinc-dependent metalloenzyme. Mol Cells 10:423-31.

19. Dengler V, McCallum N, Kiefer P, Christen P, Patrignani A, Vorholt JA, Berger-Bächi B, Senn MM. 2013. Mutation in the C-di-AMP cyclase dacA affects fitness and resistance of methicillin resistant Staphylococcus aureus. PLoS One. 8(8):e73512.

20. Xiong Y, Steitz TA. 2006. A story with a good ending: tRNA 3'-end maturation by CCA-adding enzymes. Curr Opin Struct Biol 16:12-17.

21. Burghard-Schrod M, Altenburger S, Graumann PL. 2020. The Bacillus subtilis dCMP deaminase ComEB acts as a dynamic polar localization factor for ComGA within the competence machinery. Mol Microbiol 113:906-922.

22. Iwasaki W, Sekine S-i, Kuroishi C, Kuramitsu S, Shirouzu M, Yokoyama S. 2006. Structural basis of the water-assisted asparagine recognition by asparaginyl-tRNA synthetase. J Mol Biol 360:329-342.

23. Roberts DP, Dery PD, Yucel I, Buyer JS. 2000. Importance of pfkA for rapid

growth of Enterobacter cloacae during colonization of crop seeds. Appl Environ Microbiol 66:87-91.
